# Thermodynamic Analysis of the Conformational Transition in Aqueous Solutions of Isotactic and Atactic Poly(Methacrylic Acid) and the Hydrophobic Effect

**DOI:** 10.3390/polym8050168

**Published:** 2016-04-28

**Authors:** Ksenija Kogej

**Affiliations:** Department of Chemistry and Biochemistry, Faculty of Chemistry and Chemical Technology, University of Ljubljana, SI-1000 Ljubljana, Slovenia; ksenija.kogej@fkkt.uni-lj.si; Tel.: +386-1-479-8538

**Keywords:** polymethacrylic acid, isotactic, conformational transition, thermodynamics, temperature dependence, hydrophobic effect, hydrophobic hydration

## Abstract

The affinity of amphiphilic compounds for water is important in various processes, e.g., in conformational transitions of biopolymers, protein folding/unfolding, partitioning of drugs in the living systems, and many others. Herein, we study the conformational transition of two isomer forms of poly(methacrylic acid) (PMA), isotactic (iPMA) and atactic (aPMA), in water. These isomers are chemically equivalent and differ only in the arrangement of functional groups along the chain. A complete thermodynamic analysis of the transition of the PMA chains from the compact to the extended form (comprising the conformational transition) in water in the presence of three alkali chlorides is conducted by determining the free energy, enthalpy, and entropy changes of the process as a function of temperature, and therefrom also the heat capacity change. The heat capacity change of the transition is positive (+20 J/K mol) for aPMA and negative (−50 J/K mol) for iPMA. This result suggests a different affinity of PMA isomers for water. The conformational transition of iPMA is parallel to the transfer of polar solutes into water, whereas that of aPMA agrees with the transfer of nonpolar solutes into water.

## 1. Introduction

An important point in investigating the solution behavior of polyelectrolytes is the relation between structure and properties. Very appropriate samples for such studies are those with the same chemical structure but different spatial orientation of functional groups along the chain, such as the three stereoregular forms of poly(methacrylic acid), PMA [1]: isotactic, syndiotactic, and the usual heterotactic (or atactic) PMA: iPMA, sPMA, and aPMA, respectively (for structures see Scheme S1 in the Supplementary Materials). The interest in PMA, a weak polyelectrolyte, was in the past related to another property, *i.e.*, the conformational transition of the chain induced by ionization of carboxyl groups [2], which places PMA side by side with biopolymers. The conformational transition entails a cooperative change of the shape of the polymer chain from a compact to a more open (extended) form. This feature has been and still is a focus of extensive investigations with both natural and synthetic polymers. The compact conformation is of a particular interest because the native conformations of biological macromolecules (e.g., globular proteins) are compact, *i.e.*, tightly packed. Such compact coils or globules, as they are often called, offer sites for incorporation of smaller nonpolar (hydrophobic) solutes like drug molecules. When a change in conformation is induced by some environmental factor (most often pH or temperature) the polymer shape changes to a more open one and the nonpolar solute is released into the aqueous medium [3]. In order to direct and control such processes, the nature of the compact conformation and the mechanism of the chain unfolding have to be characterized.

One of the prerequisites for the occurrence of a cooperative change in conformation is the presence of hydrophobic side groups on the chain and/or the ability to form hydrogen (H) bonds. In the case of PMA, the hydrophobic groups responsible for the conformational transition are the methyl ones. In addition, hydrogen bonds can form between carboxyl, COOH, groups intra- and also intermolecularly, the latter leading to association/aggregation between chains. The ability to form intermolecular associates between aPMA chains was mostly excluded in the past. However, it has been demonstrated by light scattering measurements [4,5,6] that this option should not be completely ignored, in particular when aPMA chains are essentially uncharged, *i.e.*, at low pH. With iPMA, intermolecular association is clearly very important and reflected in several properties [2,5,6,7,8,9,10,11,12,13]: (i) iPMA is insoluble in water below a certain critical degree of neutralization, α_N_, of carboxyl groups (α_N_ < 0.2); (ii) it is a weaker acid in comparison with aPMA over the whole region of α_N_ values; (iii) the conformational transition of the isotactic chain is irreversible, denoting that charging (neutralization) and discharging (protonation) of the chain proceed along different paths (intermediate states); and, last but not least, (iv) iPMA chains are strongly associated/aggregated even at nonzero α_N_ values, *i.e.*, little above the solubility limit at α_N_ ≈ 0.2 [5,6]. The most obvious reason on the molecular level for this clearly different affinity of PMAs for water is the stereoregularity of iPMA or, *vice versa*, the random structure of aPMA.

Our purpose in this contribution is to explore the molecular reasons for these well manifested differences between iPMA and aPMA by using the classical thermodynamic approach. We perform potentiometric and calorimetric measurements to determine the standard free energy, Δ*G*_tr_^Ө^, and enthalpy, Δ*H*_tr_, changes and therefrom the entropy, Δ*S*_tr_, change associated with the transition of the chain from the uncharged to the (completely) charged state. These measurements are performed in the presence of different alkali chlorides, XCl (X = Li, Na, Cs). In addition, measurements are carried out at different temperatures (15, 25, 45 °C) in order to evaluate also the heat capacity change, (Δ*c*_p_)_tr_, of the transition. Thermal properties, in particular the sign of (Δ*c*_p_)_tr_, are namely the fingerprint of the hydrophobic effect [14,15,16,17], which drives many complex processes in chemistry and biology.

## 2. Materials and Methods

### 2.1. Materials

Isotactic poly(methacrylic acid), iPMA, was prepared by hydrolysis of the isotactic poly(methylmethacrylate), iPMMA (Aldrich Chem. Co., St. Luis, MO, USA; weight-average molar mass *M*_w_ = 6900 kg/mol; polydispersity index PDI = 8.63) following procedures reported in the literature [10,11,18]. First, the tacticity of the starting iPMMA was checked by NMR spectroscopy. The ^1^H NMR spectrum of the polymer in CDCl_3_ (Figure S1a in the Supplementary Materials) showed the following stereoregular composition: 92% of isotactic and 4% each of syndiotactic and atactic triads. This result is in excellent agreement with the data provided by the supplier (isotacticity of 91.6%). The degree of hydrolysis of the ester groups for the hydrolyzed product, iPMA, was determined from the ^1^H NMR spectrum of iPMA in D_2_O and was greater than 99% (Figure S1b, Supplementary Materials). NMR spectroscopy was performed with Bruker Avance DPX 300 (Billerica, MA, USA) and Varian VXR 300 (Palo Alto, CA, USA) spectrometers, both operating at 300 MHz. The repeat unit of PMA and typical triad sequences of various stereoisomer forms of PMA (isotactic, atactic, and syndiotactic) are shown in Scheme S1 on the Supplementary Materials.

Dialysis (dialysis membranes with *M*_w_ cutoff of 10,000 Da) was used for final purification of iPMA. The polymer, which was in the sodium salt form after hydrolysis (pH ≈ 8–9), was first dialyzed against water to remove excessive NaOH and then against 0.02 M HCl to exchange Na^+^ for H^+^ ions. At α_N_ = 0, iPMA precipitated from water. The dialysis was continued against single and triple distilled water by exchanging it several times. The purified precipitate was filtered, dried by lyophilization (Heto HETOSSIC, Type CD 2.5; Heto-Holten A/S, Allerød, Denmark), and stored in a desiccator. The *M*_w_ and PDI of the final iPMA were not analyzed. However, the obtained product was analyzed by dynamic light scattering in order to determine the overlap concentration, c*, under the relevant experimental conditions used in this paper (see below). These data (see Table S1 in the Supplementary Materials and some c* values reported below) suggest that the transformation of the ester (iPMMA) into the acid form (iPMA) resulted in considerable reduction of *M*_w_ and PDI.

The atactic form of PMA, aPMA, was obtained by direct polymerization of methacrylic acid, MA. It was characterized previously by light scattering measurements (3DDLS, LS Instruments, Fribourg, Switzerland and size exclusion chromatography [4], providing the following values for *M*_w_ and PDI: *M*_w_ = 131 kg/mol and PDI = 2.44. To determine the tacticity, aPMA was methylated with diazomethane and analyzed by ^1^H NMR in CDCl_3_ (for the NMR spectrum see Figure S2, Supplementary Materials). The sample is predominately syndiotactic: it contains around 49% of syndiotactic, 39% of heterotactic, and 12% of isotactic triads. This is a typical triad content when PMA is obtained by direct polymerization of MA. Before use, aPMA was purified by dialysis, concentrated by vacuum distillation and stored as a concentrated stock solution in the refrigerator. The concentration of the stock solution was determined by potentiometric neutralization titration with a standardized NaOH solution (Merck, p.a.; Darmstadt, Germany) using a combined electrode InLab 406 from Mettler-Toledo (Griefensee, Switzerland) and a pH meter MA 5740 from Iskra (Ljubljana, Slovenia).

### 2.2. Preparation of Solutions

All experiments were performed in water in the presence of 0.01 M alkali chlorides, XCl (X = Li, Na, Cs) and at 15, 25, and 45 °C, if not otherwise specified. The concentration of PMA isomers, *c*_p_, was expressed in moles of COOH groups per volume and was equal to 0.01 mol/L (=M; determined at 25 °C) or 0.86 g/L for the acid form with the degree of neutralization α_N_ = 0. Parameter α_N_ is defined herein as $\alpha_{N}=c_{{\text{OH}}^{-}}/c_{p}\;$and equals zero when no hydroxide ions have been added to the polyacid solution, *i.e.*, when COOH groups on PMA are not neutralized by XOH. Later, we also define the degree of ionization, α, which is needed for the treatment of potentiometric titration curves and differs from α_N_ (for details see Section 2.3.1). The employed PMA concentration (0.01 mol/L) is well below c* as estimated from the hydrodynamic radius, *R*_h_, of iPMA and aPMA chains in 0.01 M NaCl. The *R*_h_ values were measured by dynamic light scattering measurements at 90° (*cf.* Table S1, Supplementary Materials). The data show that the lowest c* is ~20 and ~2 g/L for iPMA and aPMA, respectively, at α_N_ = 0.2. The small size of iPMA chains (*R*_h_ ≈ 20–50 nm) agrees with the presumption on considerable *M*_w_ lowering as a result of hydrolysis and dialysis.

Since iPMA is not soluble in water at α_N_ = 0 (it only dissolves when α_N_ > 0.2 [4,5,6]), the starting solution with α_N_ > 0.5 was first prepared and potentiometric and calorimetric measurements were performed in the reverse direction, *i.e.*, as protonations of the ionized COO^−^ groups instead of neutralizations of the uncharged COOH groups (see detailed description below).

In the iPMA case, a weighed amount of solid polymer was suspended in 0.01 M XCl. A calculated amount of standardized 1 M XOH solution was slowly, in small volume increments and under nitrogen atmosphere, added to the suspension in order to obtain the desired initial α_N_ value. The stock solution was diluted with a suitable 0.01 M XCl to the final concentration (*c*_p_ = 0.01 M) for the measurements. The precise α_N_ and *c*_p_ values in these stock solutions were determined by titrating the sample with 0.1 M NaOH (in the direction of increasing α_N_) and with 0.1 M HCl (in the direction of decreasing α_N_).

Although aPMA dissolves in aqueous solutions at α_N_ = 0, the reactions in the calorimeter were performed as protonations, similar to the iPMA case. Therefore, the same procedure for solution preparation was used.

### 2.3. Methods

#### 2.3.1. Potentiometric Titrations

Potentiometric titrations were performed by using a combined electrode InLab 406 from Mettler-Toledo (Griefensee, Switzerland ). Before the measurements, the electrode was calibrated at 25 °C with two aqueous buffer solutions having pH values of 6.865 and 9.18 at this temperature. The standardized aqueous XOH was first added to the PMA solution in the titration cell in order to achieve α_N_ = 1. Then the solution was retitrated with a standardized aqueous HCl solution with a concentration of 0.1 M in the direction of decreasing α_N_ by using a micro-syringe burette. The pH was measured with a pH meter Iskra MA 5740 (Ljubljana, Slovenia) after each addition and sufficient equilibration time. The stability criterion for taking a reading was d*E*/d*t* = 0.1 mV/30 s. At low pH (low α_N_) values, this may have taken up to 1 h. All titrations were carried out under nitrogen atmosphere. In the iPMA case, the nitrogen blanket was maintained over the sample to avoid foaming, which was taking place in iPMA solutions when α_N_ dropped below ~0.25.

Instead of the pH *versus* α_N_ curves, the potentiometric results are usually presented as p*K*_app_ *versus* α curves, where α is the degree of ionization and p*K*_app_ is the negative logarithm of the apparent ionization constant, both obtained from the measured pH:

(1) $$pK_{\text{app}}=\text{pH}+\text{log}\frac{1-\alpha }{\alpha }$$

The degree of ionization is related to the degree of neutralization by the expression

(2) $$\alpha =\alpha_{N}+\frac{\left[H^{+}\right]-\left[{\text{OH}}^{-}\right]}{c_{p}}$$

which follows from the electro-neutrality condition. [H^+^] and [OH^−^] are the activities of hydrogen and hydroxyl ions, respectively, calculated from the measured pH. The difference between α and α_N_ is significant only for α_N_ < 0.1 and vanishes for higher values.

Titration curves of the type p*K*_app_ = *f*(α) have been used before to evaluate the standard free energy change of a transition, Δ*G*_tr_^Ө^, for polymers undergoing the conformational transition:

(3) $$\Delta G_{\text{tr}}^{Ө}=2.303\;RT\;\int^{\text{​}}\left[pK_{\text{app}}\left(a\right)-pK_{\text{app}}\left(b\right)\right]d\alpha$$

It has been shown [19,20] that this Δ*G*_tr_^Ө^ value can be evaluated as the integral taken over the following charging-discharging cycle:

(4) $$\text{compact form}\left(\alpha =0\right)\overset{\text{path a}}{\to }\text{charged coil form}\left(\alpha \right)\overset{\text{path b}}{\to }\text{uncharged coil form}\left(\alpha =0\right)$$

The integral in Equation (3) is the area bounded by so called “a” and ”b” state curves in the low α region. The ”a” state curve corresponds to the experimentally determined $pK_{\text{app}}\left(a\right)=f\left(\alpha \right)$ data and is affected by the cooperative change in chain conformation. The ”b’” state curve ($pK_{\text{app}}\left(b\right)$ *versus* α) is an assumed curve that applies to the case where no cooperative conformational transition of the chain takes place during the protonation (discharging) of the polymer. To obtain the ‘”b” state curve for aPMA, extrapolation procedures proposed by Leyte and Mandel were used [12,21,22]. The final result of these procedures is shown in Figure S3 (Supplementary Materials) for aPMA in aqueous 0.01 M LiCl and NaCl solutions at 25 °C.

In the iPMA case, the procedure to calculate the Δ*G*^Ө^ values using Equation (3) was different due to the interference of the conformational transition with intermolecular association, which is eventually followed by precipitation of the polymer from solution. The “a” state curve again corresponds to the experimentally determined ${\text{pK}}_{\text{app}}\left(a\right)=f\left(\alpha \right)$ data. In the iPMA case, this curve is affected by both the change in chain conformation and also by intermolecular association and subsequent precipitation of the polymer. The “b” state curve (the case with no conformational transition and eventual other processes) was determined for iPMA in the same way as for aPMA (see above). In order to evaluate separately the contributions of the conformation transition and precipitation (and/or association), a hypothetical curve neglecting these two processes was constructed in the region of low α values (0.0 ≲ α ≲ 0.25) by taking into consideration the experimental $pK_{\text{app}}=f\left(\alpha \right)$ curve for iPMA in the region 0.15 ≲ α ≲ 0.3 and the shape of the curve for aPMA for α < 0.25. This curve is denoted as the ‘c’ state curve in Figure S3 (Supplementary Materials). Figure S3 shows the final results of these extrapolation procedures for both PMAs under the same experimental conditions (*i.e.*, in 0.01 M LiCl and NaCl at 25 °C). For comparison, the titration curve for poly(acrylic acid), PAA, which is not subjected to the conformational transition, is also shown in Figure S3 for the case of 0.01 M NaCl. Curves for iPMA and aPMA at all investigated conditions are reported in the Results Section. The respective integration procedures to obtain the Δ*G*^Ө^ values for iPMA using Equation (3) are described in detail in the Supplementary Materials. In this case, integrals in Equation (3) are taken over two cycles as denoted by Equations (S2a) and (S2b).

#### 2.3.2. Isothermal Titration Calorimetry

**Figure 1:**
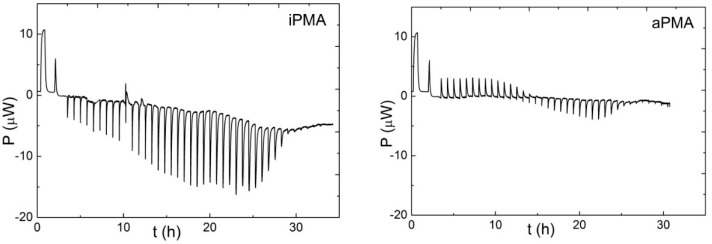
Raw ITC data (power, *P*, in μW) obtained from injections of 5 μL aliquots of 0.06 M HCl into 2 mL of 0.01 M iPMA (**left**) or aPMA (**right**) in 0.01 M aqueous LiCl solutions at 25 °C. The first peak in both thermograms corresponds to static and the second one to dynamic calibration. Subsequent peaks are due to the protonation reaction.

Isothermal Titration Calorimetry (ITC) was used to determine the ionization enthalpies of both PMA isomers. ITC is a technique that measures the heat absorbed or released upon step-wise additions of one solution into the other. ITC measurements were carried out in a TAM 2277 calorimeter (Thermometric AB, Jarfalla, Sweden). The ionization enthalpies, Δ*H*_ion_, of i- and aPMA were determined as protonation enthalpies, Δ*H*_prot_. The calorimeter cell was filled with 2 mL of 0.01 M PMA previously neutralized with a desirable 1 M XOH solution to some initial α value, which was well above the α-region of the conformational transition (see above). The solution was then titrated back to α = 0 with a 0.06 M HCl solution. The titrant (HCl solution) was added into the cell in small volume increments (usually 5 μL) with a motor run syringe up to the total volume of 250 μL. Care was taken that all solutions were degassed prior to measurements. Examples of raw thermograms obtained for iPMA and aPMA in 0.01 M LiCl at 25 °C are shown in Figure 1. An exothermic reaction results in a negative and an endothermic one in a positive peak signal (note that Δ*H*_ion_ is the negative value of Δ*H*_prot_!). Integration of the peaks provides the heat per injection. In order to calculate the value of this heat, the calorimeter was calibrated by passing a known electric current through an electric heater with a known electric power usually for 1800 s (calibration corresponds to the first two peaks in the thermogram). Subsequent peaks in Figure 1 are due to the protonation reaction in the cell. The measured heat effects were corrected for the enthalpies of dilution of PMA and HCl. The heats of dilution of HCl and both PMAs were measured in a separate experiment and were found to be negligible in comparison with Δ*H*_prot_.

#### 2.3.3. Isothermal Batch Calorimetry

Isothermal Batch Calorimetry (IBC) was used to measure the enthalpies of dilution, Δ*H*_D_, of iPMA and aPMA at 25 °C. For this purpose, an LKB 10700 Batch Calorimeter (Bromma, Sweden) was used. One compartment of the calorimeter was filled with 2 mL of iPMA (aPMA) solution with a fixed α_N_ and initial concentration (*c*_p_ = 0.065 M) value and the other one with 2 mL of the solvent. The concentration *c*_p_ = 0.065 M was chosen for comparison reasons, following similar calorimetric measurements for poly(acrylic acid), PAA, and PMA reported in the literature [23]. We also followed conditions used in that study by performing experiments in water with no added electrolyte, which has no effect on the general conclusions resulting from these measurements.

**Figure 2:**
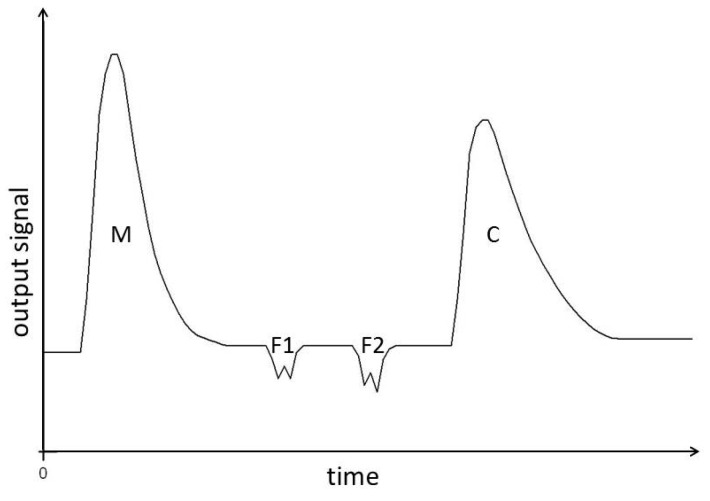
Example of an output signal from a batch calorimeter: peak designated by M represents the heat effect due to dilution of aqueous iPMA solution with water by a factor of 2 (*T* = 25 °C), peak C corresponds to calibration and peaks F1 and F2 are heat effects due to shearing of solutions in the calorimeter (see text).

Upon mixing the solutions in the calorimeter, which is achieved by rotating the calorimeter unit, the solution was diluted to half concentration. The signal from the calorimeter was amplified with a 182 Sensitive Digital Voltmeter from Keithley (Cleveland, OH, USA). After each experiment, the calorimeter was calibrated by passing an electric current *I* through an electric resistance with resistivity *R* for a known time *t*. The heating time was chosen such that the effect due to heating was comparable to that due to dilution. An example of an IBC measurement is shown in Figure 2 for iPMA. Two small peaks (F1 and F2) are the heat effects due to shearing of the solutions during mixing. These corrections were subtracted from the total heat effect. The uncertainty of the calorimetric measurements is around 10%.

## 3. Results

### 3.1. Potentiometric Titrations

**Figure 3:**
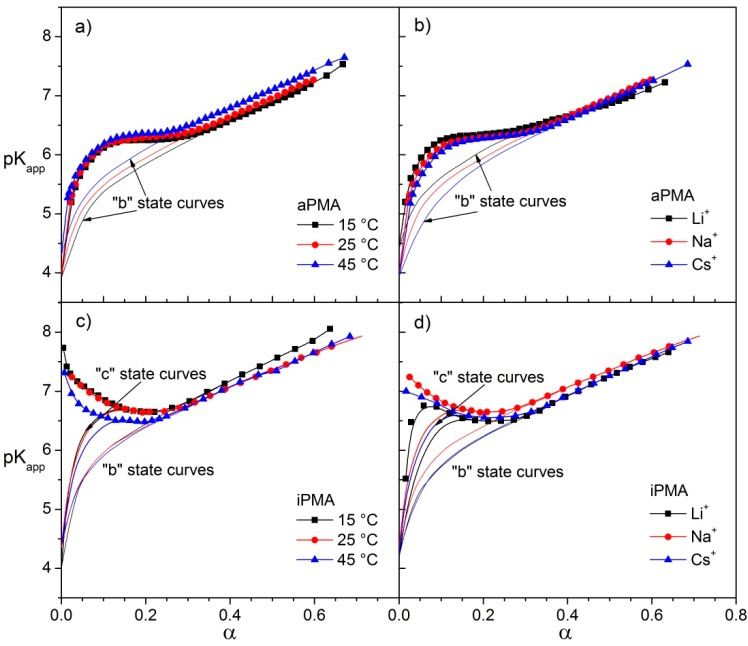
Potentiometric titration curves (p*K*_app_ *versus* α) for aPMA (**a**,**b**) and iPMA (**c**,**d**); left panel: effect of temperature (*T* = 15, 25, 45 °C) in 0.01 M NaCl; right panel: effect of counterion in 0.01 M XCls (X = Li, Na, Cs) at 25 °C. Filled symbols (together with the lines) are the experimental data (“a” state curves), dotted lines are the “b” state curves and solid lines are the “c” state or hypothetical curves for iPMA. For details on how the “b” and “c” state curves were obtained see text and Supplementary Materials.

The potentiometric titration curves (p*K*_app_ *versus* α) are shown in Figure 3 for both PMAs and for all investigated alkali chlorides and temperatures. The atactic PMA chain is known to undergo a cooperative change in conformation in aqueous solutions from a compact coil to an extended chain in an approximate α region of 0.15 ≲ α ≲ 0.3, which can be unambiguously seen in potentiometric titration curves in Figure 3a,b as the plateau with approximately constant p*K*_app_ values. This α region depends somewhat on conditions (added salt and temperature). The isotactic PMA chain is also subjected to the cooperative change in conformation; however, it is not possible to define precisely and unambiguously the limits of this event from potentiometric curves in Figure 3c,d. Similarly to aPMA, a plateau is indicated when α decreases (below α ≈ 0.3), but for α ≲ 0.2 most curves show an upward turn (an exception is the curve in LiCl). This can be explained by taking into account aggregation and precipitation of iPMA that interfere with the change in conformation at low α.

Data in Figure 3a show that p*K*_app_ values for aPMA increase with increasing temperature, but not significantly. At the same time, p*K*_app_ increases in the direction from Cs to Li. The effect of ions is more obvious in the region of low α values. On the other hand, the behavior is just the opposite with iPMA: p*K*_app_ values decrease with increasing temperature (Figure 3c) and are the lowest in the presence of Li ions (Figure 3d). Also, the sequence with respect to Na and Cs is switched. These observations are in line with the calculated Δ*G*_tr_^Ө^ values and will be commented on in more detail in the following.

As discussed above, the upward turn of p*K*_app_ values for iPMA in the low α region can be attributed to association and concomitant precipitation of the isotactic polymer. This feature is clearly seen in curves referring to iPMA in the presence of NaCl (at all temperatures) and CsCl. In the presence of LiCl, however, the shape of the p*K*_app_ *vs.* α curve for iPMA resembles the one for aPMA. Yet, instead of a plateau (aPMA), a saddle is indicated in the p*K*_app_ values in the region 0.05 ≲ α ≲ 0.25, followed by a steep decrease of p*K*_app_ for α ≲ 0.05, just as in the aPMA case. No precipitation could be detected visually during the retitration (*i.e.*, the backward titration with HCl; see Materials and Methods) of iPMA in 0.01 M LiCl, although the retitration lasted several hours. On the basis of this it was presumed that the titration curve in the presence of LiCl at low α and low polymer concentration (*c*_p_ = 0.01 M) is mostly affected by intermolecular association of iPMA chains; the contribution of precipitation can be neglected.

The curves in Figure 3 (and also in Figure S3) were used to calculate Δ*G*_tr_^Ө^ from Equation (3), as explained in the Materials and Methods. In general, this Δ*G*_tr_^Ө^ value includes contributions of all possible processes that occur in PMA solutions upon charging (or discharging) of the polymer chain. These are the change in chain conformation, Δ*G*_conf_^Ө^, and eventual intermolecular association and/or precipitation. Because the last two events are connected and partly occur simultaneously, they will be denoted with a joint term Δ*G*_ass_^Ө^:

(5) $$\Delta G_{\text{tr}}^{Ө}=\Delta G_{\text{conf}}^{Ө}+\Delta G_{\text{ass}}^{Ө}$$

We assume that in the aPMA case the calculated Δ*G*_tr_^Ө^ value only includes the contribution from the conformational transition, therefore we put Δ*G*_tr_^Ө^ ≡ Δ*G*_conf_^Ө^. This seems to be a reasonable approximation when treating the potentiometric titration curves. First, aPMA does not precipitate from solution at α = 0. But it could still be subjected to inter-molecular association leading to a non-zero Δ*G*_ass_^Ө^ term. Indeed, when self-ionization of carboxyl groups on aPMA is completely suppressed (for example by the addition of HCl) non-negligible intermolecular association was detected by light scattering [4,5,6,7]. However, the association ceases abruptly when α is increased above 0 and may have some (small) effect on the titration curve only in the region close to α = 0. Consequently, its eventual contribution to the total Δ*G*_tr_^Ө^ value is likely to be small and was herein neglected (Δ*G*_ass_^Ө^ ≈ 0). The calculated Δ*G*_conf_^Ө^ values for aPMA are reported in Table 1.

Conversely, all three processes, *i.e.*, the change in chain conformation, intermolecular association, and precipitation, contribute to the total Δ*G*_tr_^Ө^ value in the iPMA case. The extrapolation procedures presented in the Materials and Methods and in the Supplementary Materials enabled the separation of the Δ*G*_conf_^Ө^ term from the total Δ*G*_tr_^Ө^ value. The remaining part that contains contributions from intermolecular association and precipitation is included in the Δ*G*_ass_^Ө^ term (*cf.* Equation (4)). The calculated Δ*G*_tr_^Ө^ (iPMA) and Δ*G*_conf_^Ө^ ≡ Δ*G*_tr_^Ө^ (aPMA) values are reported in Table 1 and Table 2. Table 2 also includes the other thermodynamic data, which will be presented in the following. The Δ*G*_tr_^Ө^ values (so as the Δ*G*_conf_^Ө^ ones) for iPMA increase in the direction Li → Na → Cs, whereas for aPMA, the dependence of Δ*G*_conf_^Ө^ on the cation is just the opposite: it increases in the direction Cs → Na → Li. The dependence on temperature is the same in both cases: the free energy changes decrease with increasing temperature.

The Δ*G*^Ө^ values can be compared with the literature data. Crescenzi *et al.* [23] have determined Δ*G*_conf_^Ө^ = 770 J/mol for so-called conventional PMA, which is generally predominately atactic, at 25 °C in the presence of NaCl. This is in excellent agreement with our value. On the other hand, Joyce and Kuruscev [24] obtained a lower figure (Δ*G*_conf_^Ө^ = 580 J/mol), but the observed temperature dependence of Δ*G*_conf_^Ө^ was therein the same as in our case. The potentiometric behavior of iPMA was studied only by Leyte *et al.* [21], who determined that the Gibbs free energy change during the irreversible titration cycle of iPMA equals, according to their interpretation, the energy dissipation of the process. They obtained a value Δ*G*_conf_^Ө^ = 744 J/mol for iPMA, which is higher than Δ*G*_conf_^Ө^ = 650 J/mol in our case. However, the polymer in that case had a considerably lower *M* (≈335.000 g/mol) and higher isotacticity (95%–98%), both of which may contribute to this difference in Δ*G*^Ө^.

### 3.2. Ionization Enthalpies

**Figure 4:**
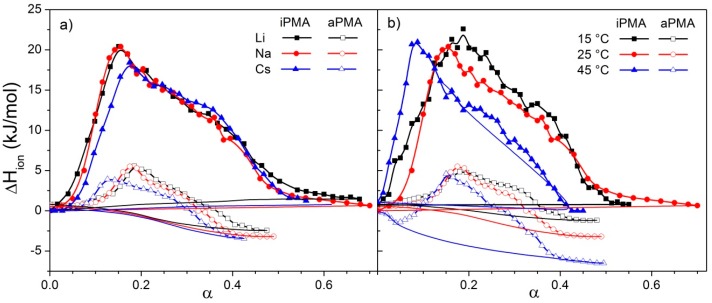
Enthalpies of ionization, Δ*H*_ion_, for iPMA and aPMA in aqueous 0.01 M XCl solutions as a function of the degree of ionization α; (**a**) *T* = 25 °C, 0.01 M XCl (X = Li, Na, Cs); (**b**) 0.01 M NaCl, *T* = 15, 25, and 45 °C. The thin solid lines (with no points) are the base lines used for integration of the peaks.

The integration of the peaks in the thermograms measured by ITC resulted in Δ*H*_ion_ values that are plotted in dependence on α in Figure 4a (for different 0.01 M XCls at 25 °C) and 4b (for different temperatures in the presence of 0.01 M NaCl) for both PMAs. Measurements for iPMA were performed for all ions at all three temperatures (these plots are shown in Figure S4, Supplementary Materials). The endothermic peak, which is seen in all curves, is considerably higher and extends over a broader α region (0.05 ≲ α ≲ 0.5) in the iPMA case than in the aPMA one (0.1 ≲ α ≲ 0.3). The ionization enthalpies of iPMA are endothermic in the whole region of α values, whereas those of aPMA become exothermic for α > 0.3. It is convenient to compare these results with the ionization enthalpies of PAA [23], which are exothermic for all α values, but decrease smoothly with increasing α, *i.e.*, they display no superimposed endothermic peak. It is known that PAA, which lacks the hydrophobic side groups on the chain (such as the methyl ones in the PMA case), does not show any cooperative change in chain conformation upon ionization of COOH groups (note that the exothermic effect accompanying the ionization of PAA is due to the hydration of charged COO^−^ groups). Clearly, the endothermic peak in Figure 4 should be attributed to this cooperative process in PMA solutions.

The area under the peaks in Figure 4 is used to calculate the enthalpy change accompanying the conformational transition, called the conformational enthalpy, Δ*H*_conf_. We use a more general term here, *i.e.*, the enthalpy of transition, and designate it as Δ*H*_tr_, in line with the designation for Δ*G*_tr_ used above. The calculation of Δ*H*_tr_ requires a base line. In the case of iPMA, where Δ*H*_ion_ values are approximately zero outside the region of the conformational transition, the constructed base line almost fits the ordinate (see the dotted lines in Figure 4). For aPMA, the Δ*H*_ion_ values clearly drop below zero for α > 0.3. The base line is constructed by taking into account points below and above the transition region. This results in similar curves (see the dashed lines in Figure 4) as reported for the ionization enthalpies of PAA [23]. The calculated Δ*H*_tr_ values are reported in Table 2 and Table 3. It should be stressed that these Δ*H*_tr_ values are not the standard ones, whereas the Δ*G*_tr_^Ө^ values are, because they are evaluated from the equilibrium constant (*cf.* Equation (3)). Similarly to Δ*G*, we note the equivalence of Δ*H*_conf_ ≡ Δ*H*_tr_ for aPMA.

**Figure 5:**
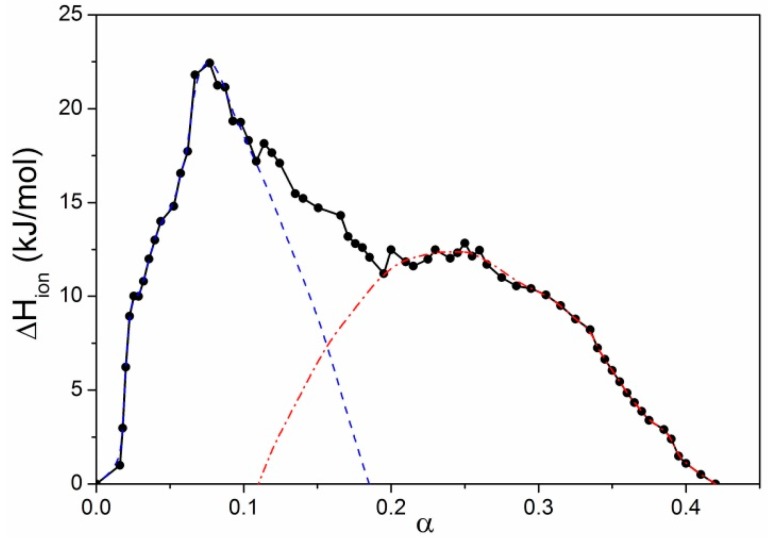
More precise calorimetric titration (Δ*H*_ion_ *versus* α curve) for iPMA in 0.01 M NaCl at 25 °C. The points are experimental values connected by the solid black line. The black and red lines designate the two superimposed peaks in the low (dashed blue line; integration of this peak resulted in the Δ*H*_ass_ value) and high (dotted red line; integration of this peak resulted in the Δ*H*_conf_ value) α region. For details on integration procedures and interpretation of these Δ*H* values see text.

The data in Table 2 and Table 3 show that Δ*H*_tr_ values are around five times higher for iPMA than for aPMA. This difference is attributed to the already discussed intermolecular association and precipitation of iPMA chains from solution. In the case of potentiometric curves, the contribution of these events could be evaluated with suitable extrapolation procedures. Also in the Δ*H*_ion_ case, a careful inspection of the calorimetric data suggests that the endothermic peak in the iPMA case may be a superposition of (at least) two peaks. This is probably the most obvious in the curve that applies to CsCl at 25 °C (Figure 4a) or to NaCl at 45 °C (Figure 4b). In order to separate the enthalpy of the cooperative conformational transition, Δ*H*_conf_, from the Δ*H*_tr_ value a more comprehensive titration was performed by titrating the iPMA solution in the calorimeter with smaller volume increments of 0.06 M HCl. This experiment was executed only at 25 °C in the presence of 0.01 M NaCl. The result is shown in Figure 5 and confirms that the ionization of iPMA is composed of at least two processes. Traced in the direction of protonation of COO^−^ groups (decreasing α, this is the direction in which the experiment was carried out), these are: the conformational transition, which is indicated in the region 0.4 ≿ α ≿ 0.2 (deconvolution resulted in the red dotted line), and association and precipitation, which are restricted to α ≾ 0.30 (for deconvolution see the blue dashed line). Separate integration of the peaks showed that conformational transition and association/precipitation contribute approximately equally (Δ*H*_conf_:Δ*H*_ass_ ≈ 1:1) to the total Δ*H*_tr_ value. Taking into account the experimental uncertainty of the technique (calorimetry), similar ratios between Δ*H*_conf_ and Δ*H*_ass_ were also obtained for other cases based on the data in Figure 4. For deconvolution of the calorimetric plots in 0.01 M LiCl and CsCl at 25 °C and in 0.01 M NaCl at 45 °C, see Figure S5 (Supplementary Materials). We thus conclude that, within the precision of the calorimetric data, the contribution of Δ*H*_conf_ and Δ*H*_ass_ to the total Δ*H*_tr_ value is approximately equal in all cases. Thus, Δ*H*_conf_ due to the conformational transition alone is still around 2.7–3.4 times higher for iPMA than for aPMA. The 1:1 splitting of Δ*H*_tr_ to the Δ*H*_conf_ and Δ*H*_ass_ terms for iPMA also suggests that both the temperature dependence and the dependence on ions of Δ*H*_conf_ alone remains the same as that of Δ*H*_tr_.

Thus, Δ*H*_tr_ (and Δ*H*_conf_) values are highest in the presence of LiCl and lowest in the presence of CsCl, irrespective of the tacticity of PMA. The nature of the cation has some minor effect on the position of the maximum. In the presence of Cs^+^ cations, it is shifted to somewhat higher α values in iPMA solutions, but just the opposite, to somewhat lower ones, in aPMA solutions. More pronounced, and at the same time contrary for iPMA and aPMA, is the effect of temperature: Δ*H*_tr_ (Δ*H*_conf_) in iPMA solutions decreases with increasing *T*, whereas it increases in aPMA solutions. This is one of the key observations that points to different affinity of the isotactic and atactic PMA towards the aqueous medium and will be discussed in detail in the Discussion.

Comparison of the measured Δ*H*_tr_ values with literature data is possible only for aPMA. Crescenzi *et al.* [23] have obtained a value Δ*H*_tr_ = 1.03 kJ/mol at a higher polymer concentration (*c*_p_ = 0.0646 M) in water without added salt at 25 °C. Taking into account the differences in experimental conditions (*c*_p_ and *c*_s_, instrumentation), the agreement with our values (Δ*H*_tr_ = 0.96–1.6 kJ/mol in 0.01 M XCls) is reasonable.

### 3.3. Dilution Enthalpies

**Figure 6:**
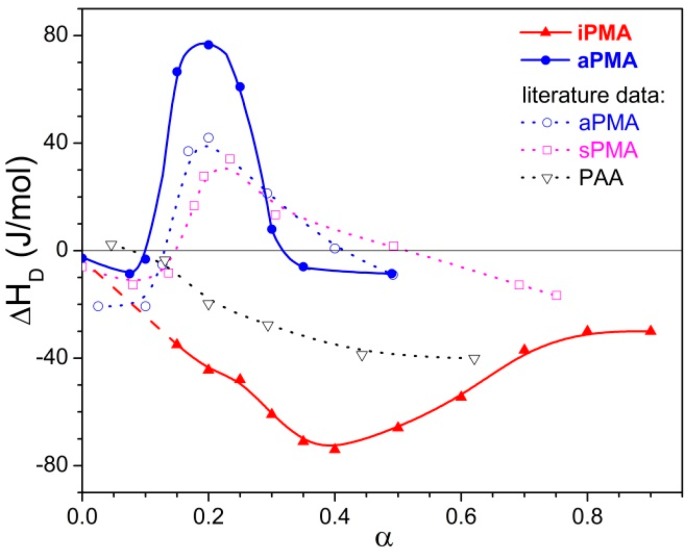
Enthalpies of dilution of iPMA and aPMA solutions in water (no added salt) at 25 °C as a function of the degree of ionization, α. The initial concentration, *c*_in_, of PMA is *c*_in_ = 0.065 M (= mol of carboxyl groups per volume) and the final one, *c*_f_, is *c*_f_ = 0.0325 M. For comparison, the literature data [23] for aPMA, sPMA, and PAA are shown (note that in Reference [23] *c*_in_ was 0.065 M and *c*_f_ was 0.0433 M).

Heat effects accompanying the dilution of iPMA and aPMA solutions in water by half (from 0.065 to 0.0325 M) are plotted in Figure 6 in dependence on α. The Δ*H*_D_ data for iPMA are limited to α ≳ 0.15 due to solubility/precipitation problems (see Materials and Methods). Nevertheless, the behavior is clearly different for both stereoisomers: Δ*H*_D_ values are negative for iPMA and (mostly) positive for aPMA, in agreement with the literature data. Crescenzi *et al.* [23] have measured the dilution enthalpies for the atactic and syndiotactic PMA(aPMA and sPMA, respectively) and also for PAA with a flow calorimeter (in contrast to the batch type used in our case). Those values are also plotted in Figure 6. It can be seen that they are mostly lower than ours, with the maximum appearing in approximately the same range of α values.

## 4. Discussion

In addition to Δ*G* and Δ*H*, a complete thermodynamic analysis of a certain process also comprises the entropy, Δ*S*, and the heat capacity, Δ*c*_p_, values. Δ*S* and Δ*c*_p_ were estimated from the measured Δ*G*_tr_^Ө^ and Δ*H*_tr_ values. To estimate the entropy change accompanying the transition, Δ*S*_tr_, at a specified temperature the standard thermodynamic equation $\Delta G_{\text{tr}}=\Delta H_{\text{tr}}-T\Delta S_{\text{tr}}$ was used. One has to keep in mind that Δ*G*_tr_^Ө^ values determined from *K*_app_ are the standard ones (applying to concentrations/activities of 1 mol/L) whereas the Δ*H*_tr_ was measured at *c*_p_ = 0.01 mol/L. However, Crescenzi *et al.* [23] have shown that Δ*H*_tr_ for aPMA is practically independent of the concentration. A value measured at any other concentration is approximately equal to the standard value. This is in agreement with our results as well (see the comparison of Δ*H*_tr_ values above) and justifies the calculation of Δ*S*_tr_ from Δ*G*_tr_^Ө^ and Δ*H*_tr_.

**Figure 7:**
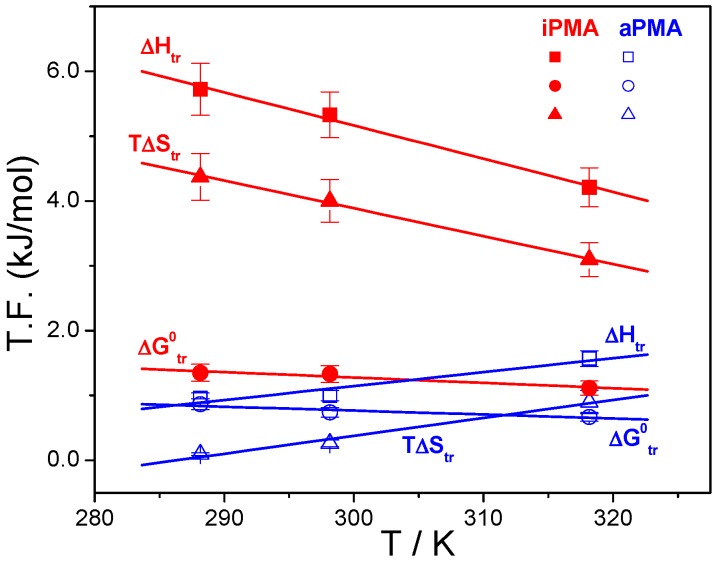
Thermodynamic functions (T.F.) accompanying the transition of iPMA and aPMA chains from the uncharged (α = 0) to the charged state (α > 0, above the conformational transition).

Results of this calculation are reported in Table 2 and plotted as a function of temperature in Figure 7, together with Δ*G*_tr_^Ө^ and Δ*H*_tr_. All Δ*S*_tr_, so as Δ*G*_tr_^Ө^ and Δ*H*_tr_, values are positive and depend on temperature and also on the added XCl. Our data for aPMA (Δ*S*_tr_ = 0.85 and 2.84 J/(Kmol) at 25 and 45 °C, respectively, in 0.01 M NaCl) can again be compared with the literature values [23]: at 25 °C (45 °C) the reported Δ*S*_tr_ values are 0.84 (2.23) and 0.71 (2.72) J/(Kmol) for aPMA and sPMA, respectively. It is well established that aPMA and sPMA display similar solution behavior. We therefore conclude that the agreement is good. The Δ*S*_tr_ values for iPMA are reported in this study for the first time, preventing any comparison with the published data.

Let us focus first on the effect of ions. For both PMAs, Δ*H*_tr_ and Δ*S*_tr_ are the lowest in CsCl solutions, whereas the effect of LiCl and NaCl may be regarded as comparable. This effect of ions can be accounted for by the so-called law of matching water affinities, LMWA [25,26,27,28], which is related to the hydration radii and enthalpies of the ions. LMWA was originally proposed to explain the well-known Hofmeister series, which is related to the specific effect that ions have on the precipitation of proteins from solutions. However, it was recently demonstrated that these effects are important not only in so-called ion-pairing phenomena but also in the interaction of ions with uncharged/neutral surfaces [29,30], for example with the acidic groups on macromolecules [30] such as the protonated carboxyl group on PMA.

The Li^+^ ion, with the smallest atomic radius, is more strongly hydrated (coordinated with more water molecules) than the Cs^+^ ion, which has a larger atomic radius. The PMA’s carboxyl, COO^−^, group is also strongly hydrated. Its hydration enthalpy is actually the closest to that of Na^+^ ions. LMWA states that the closer the hydration enthalpy of ions the stronger their mutual interaction (*i.e.*, the formation of an ion pair between X^+^ and COO^−^), through which ions lose their hydration shell and form so-called contact ion pairs (CIP). An opposite case is the solvent shared ion pair (SIP) characteristic of the combination of two large ions that do not dehydrate (an example is the pair Cs^+^/F^−^). Thus, Li^+^ and Na^+^ ions bind more strongly to the PMA’s carboxyl groups than the Cs^+^ ones, and form CIPs. The attraction of larger Cs^+^ ions with the carboxyl group is less pronounced since it involves a shared solvation shell (SIP). How does this affect the Δ*H*_tr_ values? Stronger binding facilitates formation of a more compact coil, which requires more energy input to stretch the chain upon ionization and leads to higher Δ*H*_tr_ values in LiCl (iPMA) or NaCl (aPMA) in comparison with CsCl solutions. The same conclusion on the effect of ions was obtained previously from light scattering studies of intermolecular association of both PMA isomers in aqueous XCl solutions at low α [6]. In that case, a reversed order of Na^+^ and Li^+^ in relation to the size of microgel-like aggregates between iPMA or aPMA chains was observed, in agreement with the present findings. Note that the aggregation refers to the low α state of PMAs. Although PMA chains at low α are only weakly charged, the LMWA seems to apply fairly well, in agreement with recent computer simulations involving a neutral protein [29] or an acidic surface with carboxyl groups [30]. The neutral protein followed the same series with respect to ion specificity as did the charged proteins [29]. It was therefore argued by the authors [29] that the cation specificity is determined mainly by the properties of the cation’s hydration water, in particular their ability to donate hydrogen bonds to carboxylate groups. Pronounced ion-specific effects were observed also in the case of acidic carboxyl groups [30], irrespective of whether they were protonated (uncharged) or not (*i.e.*, charged). Upon increasing pH, a pH-dependent reversal of the Hofmeister series was observed in that case, which was attributed to the change in affinities of cations for the protonated and deprotonated carboxyl groups [30]. The authors suggested that these effects arose from direct ion-surface (charged or uncharged) and indirect hydration-related interactions. To conclude, the phenomena taking place during titration of PMA in relation to ion-specificity are highly complex and may present a challenge for a separate study.

The effect of ion size/hydration on Δ*S*_tr_ is more difficult to explain due to several, sometimes contradicting, contributions. However, the loss of hydration water upon binding (see above) definitely makes an important contribution to the positive Δ*S*_tr_ value. When the PMA chain is gradually charged along the titration path, X^+^ starts to strongly interact with COO^−^, *i.e.*, they bind electrostatically to the negatively charged chain. Because Li^+^ and Na^+^, together with COO^−^, lose more water molecules through this interaction than does Cs^+^, the entropy increase due to dehydration is largest in the presence of LiCl and NaCl. It is frequently the case that the contribution of hydration/dehydration prevails over other effects. Another important effect is the electrostatic binding of X^+^ to the polyion, which leads to a decrease in the number of particles in solution and makes a negative contribution to the overall Δ*S* change.

Let us recall at the end that the total Δ*G*_tr_^Ө^ values show an opposite trend when comparing iPMA and aPMA: they increase for iPMA but decrease for aPMA in the direction Li → Na → Cs and are higher for iPMA. Because precipitation presents a considerable contribution to Δ*G*_tr_^Ө^ for iPMA, further discussion on this point would be too speculative. However, it is clear that PMA solutions display a typical case where the high, and positive, Δ*H*_tr_ and Δ*S*_tr_ values compensate for each other and result in a rather weak dependence of Δ*G*_tr_^Ө^ on temperature. Δ*G*_tr_^Ө^ values alone can therefore not be used to appraise the pronounced differences in the solution behavior of iPMA and aPMA, which was sometimes attempted in the past.

The dependence of Δ*H*_tr_ and Δ*S*_tr_ on temperature points to differences in the interaction of iPMA and aPMA with water and reflects a dissimilar state of isotactic and atactic chains in aqueous solutions, in particular at low α. The exact opposite temperature dependence of Δ*H*_tr_ and Δ*S*_tr_ suggests that they have different affinity for water. It is generally accepted that the temperature dependence of Δ*H*_tr_ and Δ*S*_tr_ is a fingerprint of the hydrophobic effect [14,15,28]. The latter is most clearly reflected in the sign of the heat capacity change, Δ*c*_p_, accompanying a certain process. In the present case, this is the transition of a weak polyelectrolyte chain from an uncharged to a charged state, leading to a designation (Δ*c*_p_)_tr_ for the heat capacity change of the transition.

The (Δ*c*_p_)_tr_ is determined by the temperature dependence of Δ*H*_tr_ (or Δ*S*_tr_):

(6) $${\left(\Delta c_{p}\right)}_{tr}={\left(\frac{d\Delta H_{tr}}{dT}\right)}_{p}=T{\left(\frac{d\Delta S_{tr}}{dT}\right)}_{p}$$

**Figure 8:**
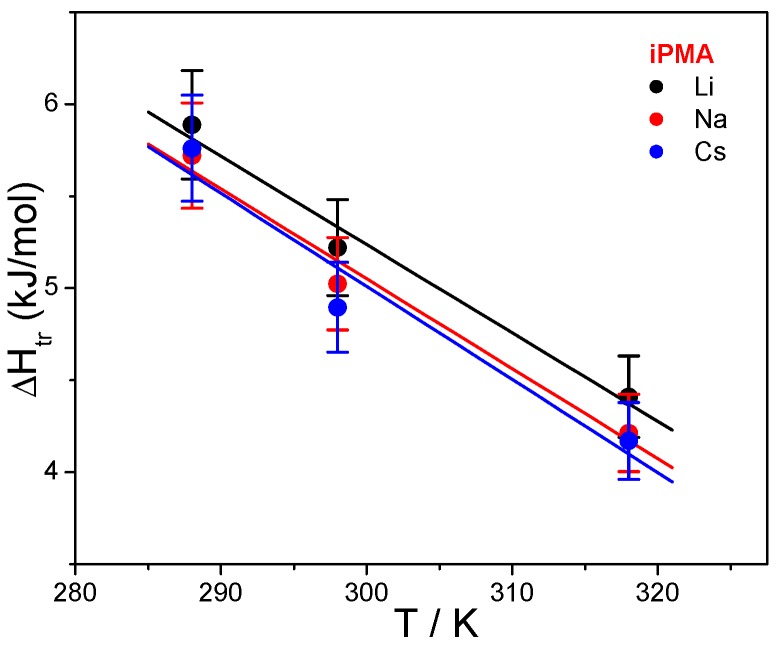
The enthalpy change, Δ*H*_tr_, accompanying the transition of iPMA chains from the uncharged to the charged state in 0.01 M aqueous XCl solutions (X = Li, Na, Cs) as a function of temperature.

Here, (Δ*c*_p_)_tr_ values were calculated from the slope of the Δ*H*_tr_ = *f*(*T*) plots. These plots can be appreciated for both PMAs in the presence of 0.01 M NaCl in Figure 7. Temperature dependencies of Δ*H*_tr_ for iPMA in the presence of all 0.01 M XCls are shown in Figure 8. For aPMA, (Δ*c*_p_)_tr_ is positive (around +20 J/Kmol), whereas it is negative for iPMA and approximately equal for all three XCls (around −50 J/Kmol). It may be noted that the (Δ*c*_p_)_tr_ values obtained from the temperature dependence of Δ*S*_tr_ are close to these, *i.e.*, +28 and −43 J/Kmol for aPMA and iPMA, respectively.

Let us recall at this point that the dilution enthalpies also show an opposite sign: they are negative for iPMA and positive for aPMA. The reason for the opposite sign of (Δ*c*_p_)_tr_ and Δ*H*_D_ of PMA isomers can be explained by proposing a different “structure” for the compact coil of iPMA and aPMA in water at low α, which entails different hydration, *i.e.*, interaction with water. In the following, the features of the compact state of iPMA and aPMA in aqueous solutions are discussed in detail.

Water is a highly structured solvent due to hydrogen (H)–bonds between water molecules. When a solute is dissolved in water, the H-bond network of pure water is perturbed. If the solute is polar, new H–bonds between water and the solute are formed, which is energetically favorable (the hydration enthalpies are usually strongly negative—exothermic). However, if the solute is non-polar (hydrophobic) it does not form H-bonds with water, but its presence gives rise to enhanced H–bond formation between water molecules themselves. This is known as the hydrophobic effect (also hydrophobic hydration). The structuring of water around a simple hydrophobic solute is sometimes described as the iceberg model. As a consequence, the transferring of simple hydrophobic solutes into water usually results in positive Δ*H* and Δ*G* values (consumption of energy due to the breaking of H-bonds). However, it has been shown [14,15,16,17,28] that the sign of Δ*c*_p_, which is determined by the temperature dependence of Δ*H* (or Δ*S*), is actually the most important factor in appraising the hydrophobic effect and hydrophobic hydration. The hydration of a non-polar solute (or its transfer into water) produces a positive and the hydration of a polar solute a negative Δ*c*_p_ value. This rule applies, for example, to protein folding/unfolding [16]. The thermal features of protein unfolding resemble those of transferring a small nonpolar solute into water.

**Figure 9:**
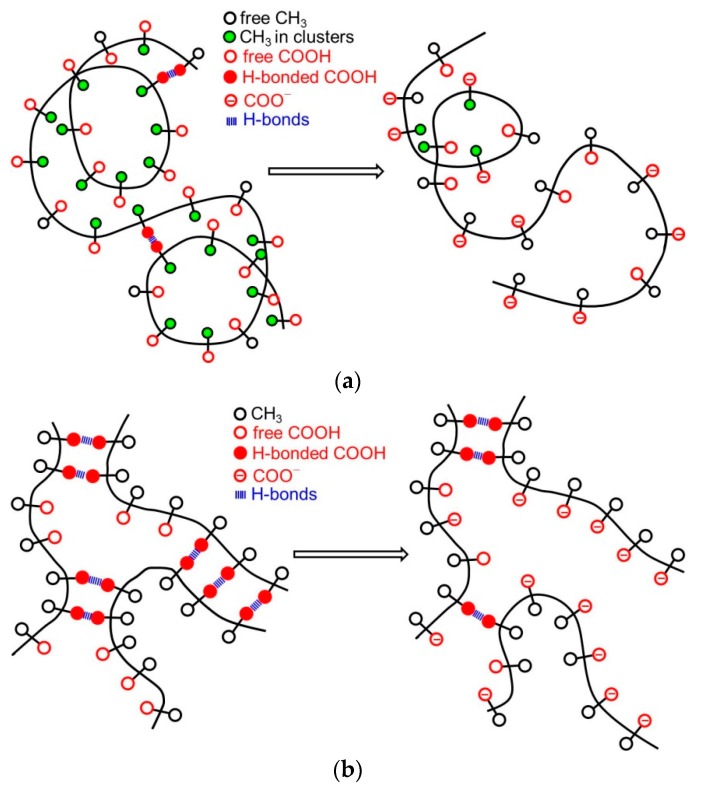
A schematic representation on the molecular level of the unfolding of the (**a**) aPMA and (**b**) iPMA chains caused by ionization of carboxyl groups or by dilution of solutions. Note that in the case of ionization, carboxyl groups attain a negative charge (which is indicated in the Figure) whereas upon dilution these groups remain uncharged or retain the same charge as in the initial state.

The positive (Δ*c*_p_)_tr_ accompanying the process of charging the aPMA chain is thus parallel to the transfer of a hydrocarbon (non-polar solute) into water [14,31] or to protein unfolding [16,31]. The Δ*c*_p_ values for protein unfolding are somewhat higher, in the range of 0.1–0.2 cal/K per gram of polymer [17] (in the same units, the (Δ*c*_p_)_tr_ for aPMA determined in this work is 0.055–0.078 cal/K per gram of the acid form of aPMA). It can be easily visualized for aPMA that due to charging and unfolding of the chain, the methyl groups that are buried inside the compact aPMA core are exposed to water, similar to the case of protein unfolding. In the latter case, it is the nonpolar amino acids that are exposed. On the other hand, the negative (Δ*c*_p_)_tr_ in the iPMA case agrees with the hydration of a typical polar solute. For example, the dissociation of acetic acid is accompanied by a strongly negative (Δ*c*_p_)_tr_ (=−142 J/Kmol [14]). Negative (Δ*c*_p_)_tr_ values stem from strong hydration of ions, which results in the loss of entropy due to the more ordered water structure in the hydration shell. It was recently demonstrated by molecular dynamics simulations [31] that the overall negative heat capacity changes (the Δ*c*_p_ values) of common salts are caused by the anion (F^−^ in that case [31]), while the cation (Na^+^ in that case [31]) actually contributes positively. The opposite sign of Δ*c*_p_ of individual ions was attributed to strong asymmetry in the orientation of water molecules around positively and negatively charged groups. Experimentally, of course, it is the sum of heat capacities that is determined; in the present approach we have no means to determine that. To return to iPMA, the negative (Δ*c*_p_)_tr_ value in this case is in apparent contradiction with the well-defined hydrophobic nature of iPMA at low α, which is reflected in its insolubility in water and in high aggregation tendency [5,6]. How can this contradiction be resolved? For this purpose, we propose the following model of unfolding the iPMA and aPMA chains in water, which is schematically presented in Figure 9. The unfolding of the chains is brought about either by ionization of carboxyl groups or by dilution of the polymer at a constant α value. The ionization undoubtedly causes a more pronounced change than dilution.

Let us start with aPMA. A schematic representation of the unfolding of the aPMA chain is shown in Figure 9a. In the compact form of aPMA, most of the hydrophobic methyl groups (open black circles) are buried inside the core, whereas the carboxyl groups (open red circles) are oriented towards the aqueous environment and thus effectively protect the methyl groups from unfavorable contact with water. Responsible for the stability of the compact form of aPMA are the short-range interactions, *i.e.*, van der Waals forces, the hydrophobic effect associated with the methyl groups and partly also H-bonds (all this is schematically captured in Figure 9a). Such picture is in agreement with the solubility of aPMA in water at α = 0 and also with low polarity of the compact core, as demonstrated through pyrene polarity ratio measurements [13]. When the aPMA chain unfolds (due to long-range repulsive electrostatic interactions), the nonpolar methyl groups from the core are exposed to the aqueous environment. This process is parallel to the transfer of a hydrophobic solute into water and therefore accompanied by a positive (Δ*c*_p_)_tr_. Note that only one aPMA chain is shown in the scheme in Figure 9a in order to keep presentation simple. However, it has to be kept in mind that aPMA chains are intermolecular, associated at α = 0 [4].

In the unfolding of iPMA chains, a qualitatively opposite process occurs that parallels the transfer of a polar solute into water. iPMA chains are much more strongly aggregated in aqueous solutions in comparison with the aPMA ones. The mode of aggregation is different as well. Light scattering measurements [4] suggest the formation of microgel-like aggregates of several iPMA chains just above the α value (α ≈ 0.25) defining the limit of its solubility in water, and that in spite of a non-negligible charge of the chains (recall again that intermolecular association of aPMA chains occurs only at or close to α = 0 [4]). Liable for this is the ordered isotactic arrangement of the functional groups, which is the basis for a very strong and cooperative hydrogen bonding between chains [5,6,11]. The methyl groups play an important role in this cooperativity by reinforcing the H-bonds between carboxyl groups. It has been shown by density functional theory calculations [32] that the strength of H-bonds is significantly increased if an electron donating group (like methyl) is bound next to the carboxyl group. In fact, such reinforcement must be expected for both aPMA and iPMA, but not for PAA. It was demonstrated by statistical mechanics [33] that the PAA chain merely extended in solution upon ionization, whereas it swelled substantially in an abrupt way, *i.e.*, in a narrow pH range, in the PMA case. It can be easily visualized that the particular orientation of carboxyl groups on the isotactic chain (always on the same side of the chain) is favorable for the cooperativeness of the H-bonding. In agreement with this, a recent study on thin iPMA films [11] showed that a high degree of order is induced when amorphous iPMA films prepared from solutions in organic solvents are immersed in water. In line with our conclusions, the strong intermolecular association was therein ascribed to cooperative hydrogen bonding between iPMA chains that could only be disturbed by solvents capable of forming strong hydrogen bonds with the solute, like DMF and DMSO.

Recently, a two-step model of the association process between iPMA chains was proposed [6,11]. A sketch showing a detail of such an associate/aggregate of several iPMA chains is shown in Figure 9b, where the H-bonded COOH groups are shown by full red circles and the parallel blue lines indicate the H-bonds between them. In such an arrangement, a large proportion of the methyl groups (open black circle) is forced to face the solvent (water). Again, such a “model” is strongly supported by pyrene fluorescence measurements [13], which pointed out that the micropolarity of the iPMA compact conformation at low α is actually higher than that of aPMA. This is due to a different “composition” of the interior of the compact form at α = 0 in comparison with aPMA; the compact conformation of iPMA is put together mainly through hydrogen bonding and thus the methyl groups are actually forced to orient toward water, whereas the core contains more COOH groups. The breaking of H-bonds between COOH groups, either due to ionization or dilution, leads to exposure of these polar groups to water. This is equivalent to the transfer of a polar solute into water and thus accompanied by a negative (Δ*c*_p_)_tr_ value, as suggested above.

To summarize: the ionization process of both iPMA and aPMA is accompanied by positive Δ*H*_ion_ (and Δ*H*_tr_) values because energy is needed to pull a hydrogen ion from the chain and to unfold the compact coil. Upon unfolding of the aPMA coil, the number of unfavorable contacts of the methyl groups with water increases. These contacts become increasingly less favorable with increasing temperature, which leads to an increase of Δ*H*_tr_ with temperature and to a positive (Δ*c*_p_)_tr_. The situation is different with iPMA. In order to ionize the isotactic chain, first the H-bonds involved in intermolecular associates/aggregates have to be broken and then the hydrogen ions can be pulled from the chain. This is the reason, on the molecular level, for higher Δ*H*_ion_ (and Δ*H*_tr_) values in comparison with those for aPMA. Furthermore, due to ionization of carboxyl groups that are involved in hydrogen bonding between iPMA chains favorable contacts between the strongly hydrated COO^−^ groups and water are established. Δ*H*_tr_ for iPMA decreases with increasing temperature because the extent of hydration is lower at higher temperatures.

## 5. Conclusions

A thorough thermodynamic analysis of the conformational transition in iPMA and aPMA solutions shows that this process is governed by the so-called hydrophobic effect in the aPMA case. Δ*H*_tr_ and Δ*S*_tr_ increase with increasing temperature. This results in a positive (Δ*c*_p_)_tr_ value, which is characteristic for the transfer of nonpolar solutes into water or to protein unfolding. These features may be related to the presence of the methyl groups in the aPMA chain.

The methyl groups are present also in the isotactic polyacid, but the behavior of the isotactic and atactic PMA is clearly different. In the iPMA case, Δ*H*_tr_ and Δ*S*_tr_ decrease with increasing *T*. This leads to the most important result of this study, *i.e.*, to the negative (Δ*c*_p_)_tr_ value in the iPMA case. The negative heat capacity change is characteristic for the transfer of polar solutes into water. The apparent contradiction of this result with the explicit hydrophobic nature of iPMA (reflected in insolubility and strong aggregation tendency) is resolved herein by proposing a model of charging and unfolding of iPMA and aPMA chains in water upon ionization or dilution. Although iPMA is clearly more hydrophobic than aPMA, the ionization (or dilution) causes deaggregation of chains followed by the exposure of hydrophilic carboxyl groups to water. On the microscopic level, this is equivalent to the transferring of a polar solute into water. The association/aggregation between iPMA chains plays a decisive role here. The opposite is the case with aPMA, where the character of the solute changes from moderately hydrophilic in the uncharged state to hydrophobic when the methyl groups come into contact with water.

## Figures and Tables

**Table 1:** Values of the standard Gibbs free energy changes, Δ*G*_tr_^Ө^ and Δ*G*_conf_^Ө^, accompanying the transition of the iPMA and aPMA chains from the compact to the extended form in aqueous 0.01 M XCl solutions (X = Li, Na, Cs) at 15, 25, and 45 °C.

X	*T*/°C	Δ*G*_tr_^Ө^ (J/mol)	Δ*G*_conf_^Ө^ (J/mol)	Δ*G*_conf_^Ө^ (J/mol) ^1^
		iPMA		aPMA
Li	25	1,060	600	864
Na	25	1,330	650	743
Cs	25	1,425	790	668
Na	15	1,350	660	865
Na	25	1,330	650	743
Na	45	1,115	470	670

^1^ note that Δ*G*_conf_^Ө^ ≡ Δ*G*_tr_^Ө^ for aPMA (see text).

**Table 2:** Values of the total standard Gibbs free energy change, Δ*G*_tr_^Ө^, and the enthalpy and entropy changes, Δ*H*_tr_ and Δ*S*_tr_, respectively, accompanying the transition of the iPMA and aPMA chains from the compact to the extended form in aqueous 0.01 M XCl solutions (X = Li, Na, Cs) at 15, 25, and 45 °C.

Polyacid	X	*T*/°C	Δ*G*_tr_^Ө^ (J/mol) ^1^	Δ*H*_tr_ (J/mol)	Δ*S*_tr_ (J/Kmol)
iPMA	Li	25	1,060	5,220	16.2
	Na	25	1,330	5,330	13.4
	Cs	25	1,425	4,900	11.7
aPMA	Li	25	864	1,070	0.69
	Na	25	743	1,000	0.85
	Cs	25	668	850	0.61
iPMA	Na	15	1,350	5,720	15.2
	Na	25	1,330	5,330	13.4
	Na	45	1,115	4,210	10.4
aPMA	Na	15	865	960	0.33
	Na	25	743	1,000	0.85
	Na	45	670	1,570	2.84

^1^ note that Δ*G*_tr_^Ө^ ≡ Δ*G*_conf_^Ө^ for aPMA (see text).

**Table 3:** Values of the enthalpy change, Δ*H*_tr_, accompanying the transition of iPMA and aPMA chains from the compact to the extended form in aqueous 0.01 M XCl solutions (X = Li, Na, Cs) at 15, 25, and 45 °C.

Polyacid	X	*T*/°C	Δ*H*_tr_ (J/mol) ^1^	X	*T*/°C	Δ*H*_tr_ (J/mol) ^1^
iPMA	Li	15	5,890	Li	15	5,890
	Na	15	5,720	Li	25	5,220
	Cs	15	5,760	Li	45	4,410
	Li	25	5,220	Na	15	5,720
	Na	25	5,330	Na	25	5,330
	Cs	25	4,900	Na	45	4,210
	Li	45	4,410	Cs	15	5,760
	Na	45	4,210	Cs	25	4,900
	Cs	45	4,170	Cs	45	4,170
aPMA	Li	25	1,070	Na	15	960
	Na	25	1,000	Na	25	1,000
	Cs	25	850	Na	45	1570

^1^ note that Δ*H*_conf_ ≡ Δ*H*_tr_ for aPMA (see text).

## Supplementary Materials

The following are available online at www.mdpi.com/2073-4360/8/5/168/s1, Scheme S1: structures of PMA isomers, Table S1: overlap concentration in PMA solutions, Figure S1: NMR spectra of iPMMA and iPMA, Figure S2: NMR spectrum of aPMMA, Figure S3: Potentiometric titration curves for iPMA and aPMA in 0.01 M LiCl and NaCl and extrapolation procedures, Figure S4: Ionization enthalpies of iPMA, Figure S5: Deconvolution of calorimetric curves for iPMA.

## References

[1] Hatada K. (1999). Stereoregular uniform polymers. J. Polym. Sci..

[2] Crescenzi V. (1968). Some recent studies of polyelectrolyte solutions. Adv. Polym. Sci..

[3] Khutoryanskiy V.V. (2007). Hydrogen-bonded interpolymer complexes as materials for pharmaceutical applications. Int. J. Pharm..

[4] Kogej K., Berghmans H., Reynaers H., Paoletti S. (2004). Unusual behavior of atactic poly(methacrylic acid) in aqueous solutions monitored by wide-angle light scattering. J. Phys. Chem..

[5] Sitar S., Aseyev V., Kogej K. (2014). Differences in association behavior of isotactic and atactic poly(methacrylic acid). Polymer.

[6] Sitar S., Aseyev V., Kogej K. (2014). Microgel-like aggregates of isotactic and atactic poly(methacrylic acid) chains in aqueous alkali chloride solutions as evidenced by light scattering. Soft Matter.

[7] Nagasawa M., Murase T., Kondo K. (1965). Potentiometric titration of stereoregular polyelectrolytes. J. Phys. Chem..

[8] Jerman B., Kogej K. (2006). Fluorimetric and potentiometric study of the conformational transition of isotactic and atactic poly( methacrylic acid) in mixed solvents. Acta Chim. Slov..

[9] Jerman B., Breznik M., Kogej K., Paoletti S. (2007). Osmotic and volume properties of stereoregular poly(methacrylic acids) in aqueous solution: Role of intermolecular association. J. Phys. Chem. B.

[10] Loebl E.M., O’Neill J.J. (1960). Solution properties of isotactic polymethacrylic acid. J. Polym. Sci..

[11] Van den Bosch E., Keil Q., Filipcsei G., Berghmans H., Reynaers H. (2004). Structure formation in isotactic poly(methacrylic acid). Macromolecules.

[12] Leyte J.C., Arbouw-van der Veen H.M.R., Zuiderweg L.H. (1972). Irreversible potentiometric behavior of isotactic poly(methacrylic acid). J. Phys. Chem..

[13] Vlachy N., Dolenc J., Jerman B., Kogej K. (2006). Influence of stereoregularity of the polymer chain on interactions with surfactants: Binding of cetylpyridinium chloride by isotactic and atactic poly(methacrylic acid). J. Phys. Chem. B.

[14] Tanford C. (1980). The Hydrophobic Effect: FORMATION of Micelles and Biological Membranes.

[15] Southall N.T., Dill K.A., Haymet A.D.J. (2002). A View of the hydrophobic effect. J. Phys. Chem. B.

[16] Dill K.A. (1990). Dominant forces in protein folding. Biochemistry.

[17] Kauzmann W. (1959). Some factors in the interpretation of protein denaturation. Adv. Prot. Chem..

[18] Klesper E., Strassil D., Regel W. (1974). Copolymer statistics during esterification of syndiotactic poly(methacrylic acid) with diazomethane. Die Makromol. Chem..

[19] Zimm B.H., Rice S.A. (1960). The helix-coil transition in charged macromolecules. Mol. Phys..

[20] Nagasawa M., Holtzer A. (1964). Helix-coil transition in solutions of polyglutamic acid. J. Am. Chem. Soc..

[21] Leyte J.C., Mandel M. (1964). Potentiometric behavior of poly(methacrylic acid). J. Polym. Sci. A.

[22] Arnold R. (1957). The titration of polymeric acids. J. Colloid Sci..

[23] Crescenzi V., Quadrifoglio F., Delben F. (1972). Calorimetric investigation of poly(methacrylic acid) and poly(acrylic acid) in aqueous solution. J. Polym. Sci..

[24] Joyce D.E., Kuruscev T. (1981). Hydrogen ion equlibria in poly(methacrylic acid) and poly(ethacrylic acid) solutions. Polymer.

[25] Collins K.D. (1997). Charge-density dependent strength of hydration and biological structure. Biophys. J..

[26] Collins K.D. (2004). Ions from the Hofmeister series and osmolytes: Effects on proteins in solution and in the crystallizazion process. Methods.

[27] Collins K., Neilson G.W., Enderby J.E. (2007). Ions in water: Characterizing the forces that control chemical processes and biological structure. Biophys. Chem..

[28] Schmid R. (2001). Recent advances in the description of the structure of water, the hydrophobic effect, and the like-dissolves-like rule. Mon. für Chem..

[29] Hess B., van der Vegt N.F.A. (2009). Cation specific binding with protein surface charges. Proc. Natl. Acad. Sci. USA.

[30] Schwierz N., Horinek D., Netz R.R. (2015). Specific ion binding to carboxylic surface groups and the ph dependence of the hofmeister series. Langmuir.

[31] Sedlmeier F., Netz R.R. (2013). Solvation thermodynamics and heat capicity of polar and charged solutes in water. J. Chem. Phys..

[32] Tao L., Han J., Tao F.-M. (2008). Correlations and predictions of carboxylic acid p*K*_a_ values using intermolecular structure and properties of hydrogen-bonded complexes. J. Phys. Chem. A.

[33] Garces J.L., Koper G.J.M., Borkovec M. (2006). Ionization equilibria and conformational transitions in polyprotic molecules and polyelectrolytes. J. Phys. Chem. B.

