# Supplementary material for: Thermodynamic Analysis of the Conformational Transition in Aqueous Solutions of Isotactic and Atactic Poly(Methacrylic Acid) and the Hydrophobic Effect
Source: Polymers (Basel). 2016 Apr 28;8(5):168. doi: 10.3390/polym8050168 (PMC6432447; doi:10.3390/polym8050168)
Supplement: Supplementary file 1 [file polymers-08-00168-s001.pdf]

Supplementary Materials: Thermodynamic Analysis of the Conformational Transition in Aqueous Solutions of Isotactic and Atactic Poly (methacrylic acid) and the Hydrophobic Effect

Ksenija Kogej

1. Schematic Representation of the PMA Repeat Unit and of Various PMA Triads

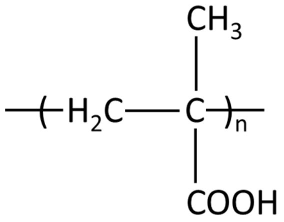

(**a**)

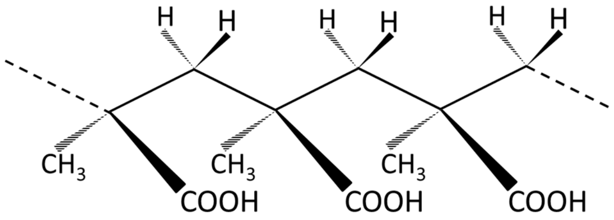

(**b**)

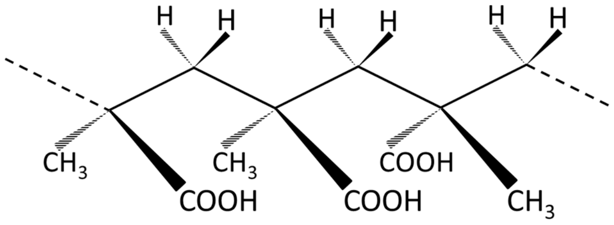

(**c**)

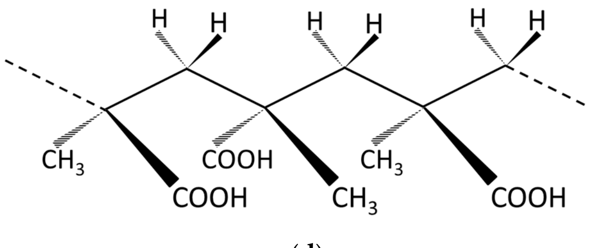

(**d**)

Scheme S1. Schematic presentation of the (**a**) repeat unit of polymethacrylic acid (PMA) and typical (**b**) isotactic (**c**) atactic and (**d**) syndiotactic sequences of three repeat units called triads. An isotactic triad is composed of two adjacent meso diads (designated as an mm triad) whereas a syndiotactic triad is composed of two adjacent racemo diads (designated as an rr triad). An atactic triad is composed of a meso (m) diad adjacent to a raceme (r) diad and is designated as an mr (or rm) triad.

Table S1. Rough estimation of the overlap concentration, c^*, of iPMA and aPMA chains in aqueous 0.01 M NaCl solutions at 25 °C as calculated from the hydrodynamic radius, R_h. The R_h was measured in 0.01 M NaCl solutions at 25 °C by dynamic light scattering at 90°.

Polymer	α_N	R_h (nm)	c^* (g/mol)
	0.20	50	20
iPMA	0.52	42	30
	1.0	20	275
	0.20	28	2.4
aPMA	0.52	17	10
	1.0	14	20

2. NMR Spectra of iPMMA, iPMA and aPMMA

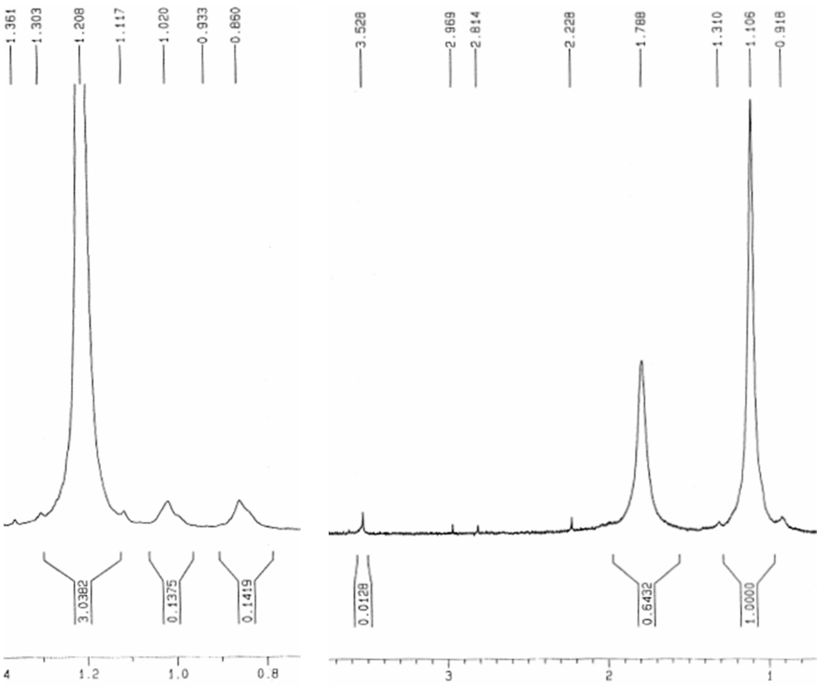

(**a**) (**b**)

Figure S1. ^{1}H NMR spectra of (**a**) iPMMA in $CDCl_3$ and (**b**) iPMA in D_2O.

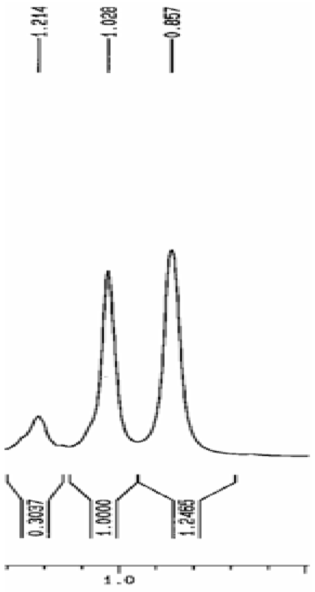

Figure S2. ^{1}H NMR spectrum of aPMMA in $CDCl_3$.

3. Treatment of Potentiometric Titration Data

Titrations curves of the type $\mathrm{p}K_{\mathrm{app}} = f(\alpha)$ are often used to evaluate the standard free energy change of a transition, $\Delta G_{\mathrm{tr}}^{\ominus}$, for polymers undergoing the conformational transition via:

$$\Delta G_{\mathrm{tr}}^{\ominus} = 2.303\ \mathrm{R}T \int [\mathrm{p}K_{\mathrm{app}}(a) - \mathrm{p}K_{\mathrm{app}}(b)] \mathrm{d}\alpha. \tag{S1}$$

The $\Delta G_{\mathrm{tr}}^{\ominus}$ value is calculated as the integral taken over the following charging-discharging cycle:

$$\text{compact form}(\alpha = 0) \xrightarrow{\text{path a}} \text{charged coil form}(\alpha) \xrightarrow{\text{path b}} \text{uncharged coil form}(\alpha = 0). \tag{S2}$$

The aPMA case. The integral in Equation (S1) is the area bounded by so called "a" and "b" state curves (*cf.* Equation (S2)) in the low α region (see Figure S3). The "a" state curve corresponds to the experimentally determined $\mathrm{p}K_{\mathrm{app}}(\mathrm{a}) = f(\alpha)$ data for aPMA and is affected by the cooperative change in chain conformation. The "b" state curve ($\mathrm{p}K_{\mathrm{app}}(\mathrm{b})$ versus α) is an assumed curve that applies to the case where no cooperative conformational transition of the aPMA chain takes place during the protonation (*i.e.*, discharging) of carboxyl groups on the chain. This curve is easily determined for aPMA following the extrapolation procedures proposed by Leyte and Mandel [12,21]. The final result of these procedures is shown Figure S3 for aqueous aPMA solutions in the presence of 0.01 M LiCl and NaCl at 25 °C.

The iPMA case. In the iPMA case, the procedure to calculate the $\Delta G^{\ominus}$ values using Equation (S1) is different due to the interference of the conformational transition with intermolecular association, which is eventually followed by precipitation of the polymer from solution (see the main manuscript). The "a" state curve again corresponds to the experimentally determined $\mathrm{p}K_{\mathrm{app}}(\mathrm{a}) = f(\alpha)$ data (see Figure S3). In the iPMA case, this curve is affected by both the change in chain conformation and also by intermolecular association and subsequent precipitation of the polymer. The "b" state curve (the case with no conformational transition) is determined in a similar way as for aPMA (see above). Then, a hypothetical curve neglecting association and precipitation is constructed in the region of low α values ($0.0 \lesssim \alpha \lesssim 0.25$) by taking into consideration the experimental $\mathrm{p}K_{\mathrm{app}} = f(\alpha)$ curve for iPMA in the region $0.15 \lesssim \alpha \lesssim 0.3$ and the shape of the curve for aPMA at $\alpha < 0.25$. This hypothetical curve is denoted as the 'c' state curve in Figure S3. In this figure, final results of these extrapolation procedures are shown for both iPMA and aPMA under the same experimental conditions (*i.e.*, in 0.01 M LiCl and NaCl at 25 °C). For comparison, the titration curve for poly(acrylic acid), PAA, which is not subjected to the conformational transition, is also shown. The respective integration to obtain the $\Delta G^{\ominus}$ values is taken over the following cycles:

$$\text{compact form}(\alpha = 0) \xrightarrow{\text{path b}} \text{charged coil form}(\alpha) \xrightarrow{\text{path a}} \text{uncharged coil form}(\alpha = 0) \tag{S3a}$$

$$\text{compact form}(\alpha = 0) \xrightarrow{\text{path b}} \text{charged coil form}(\alpha) \xrightarrow{\text{path c}} \text{uncharged coil form}(\alpha = 0) \tag{S3b}$$

and results in the total $\Delta G_{\mathrm{tr}}^{\ominus}$ term (cycle along paths "b" and "a"; *cf.* Equation (S3a)) and in the $\Delta G_{\mathrm{conf}}^{\ominus}$ (the conformational free energy: cycle along paths "b" and "c"; *cf.* Equation (S3b); note that the "c" state curve is absent in the aPMA case!). The significance of $\Delta G_{\mathrm{tr}}^{\ominus}$ and $\Delta G_{\mathrm{conf}}^{\ominus}$ terms is discussed in more detail in the main manuscript.

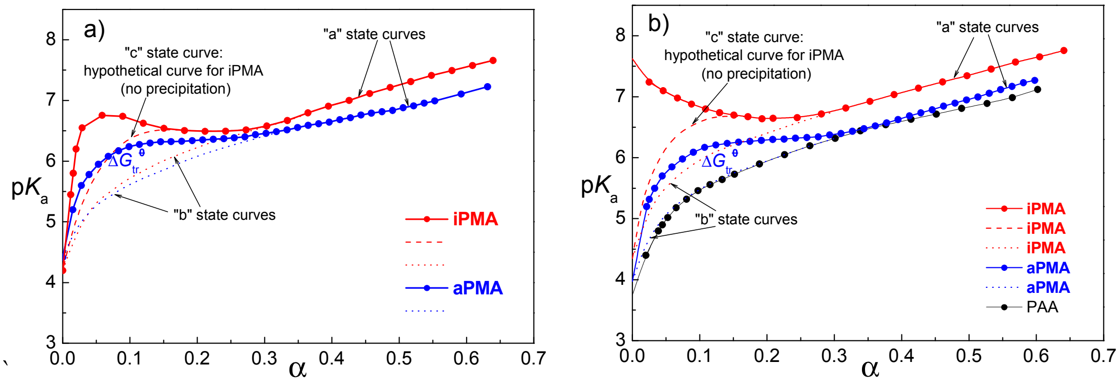

Figure S3. Potentiometric titration curves (pK_{app} versus α) for iPMA and aPMA in (**a**) 0.01 M aqueous LiCl and (**b**) NaCl solutions at 25 °C. Filled symbols are experimental data ("a" state curves), dotted lines are the "b" state curves and the dashed red line is the "c" state or the hypothetical curve for iPMA. For details on how these curves were obtained see text. Figure S3b) shows also the potentiometric titration curve for polyacrylic acid, PAA (in black).

4. Ionization Enthalpies of iPMA

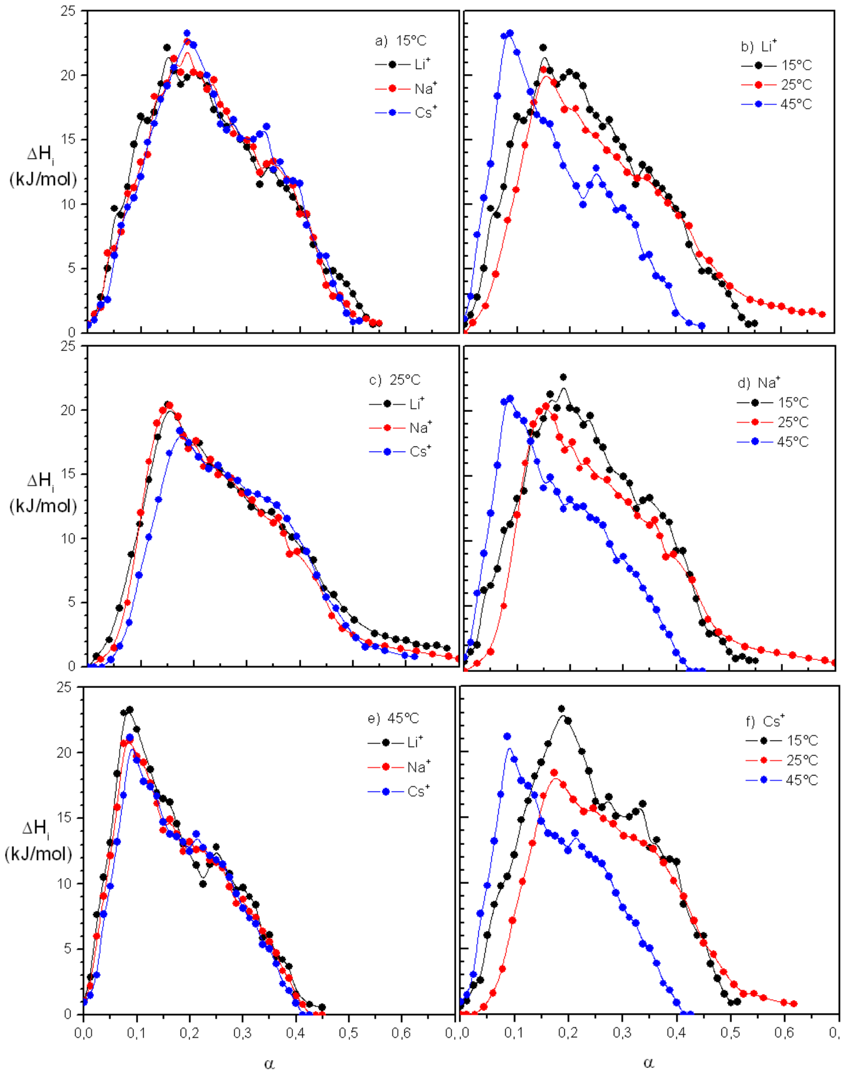

Figure S4. The effect of added alkali chloride (0.01 M XCl) at (**a**) T = 15 °C, (**c**) T = 25 °C and (**e**) T = 45 °C and the effect of temperature in (**b**) 0.01 M LiCl, (**d**) 0.01 M NaCl and (**f**) 0.01 M CsCl on enthalpies of ionization, ΔH_{ion}, of iPMA.

5. Deconvolution of Calorimetric Curves for iPMA

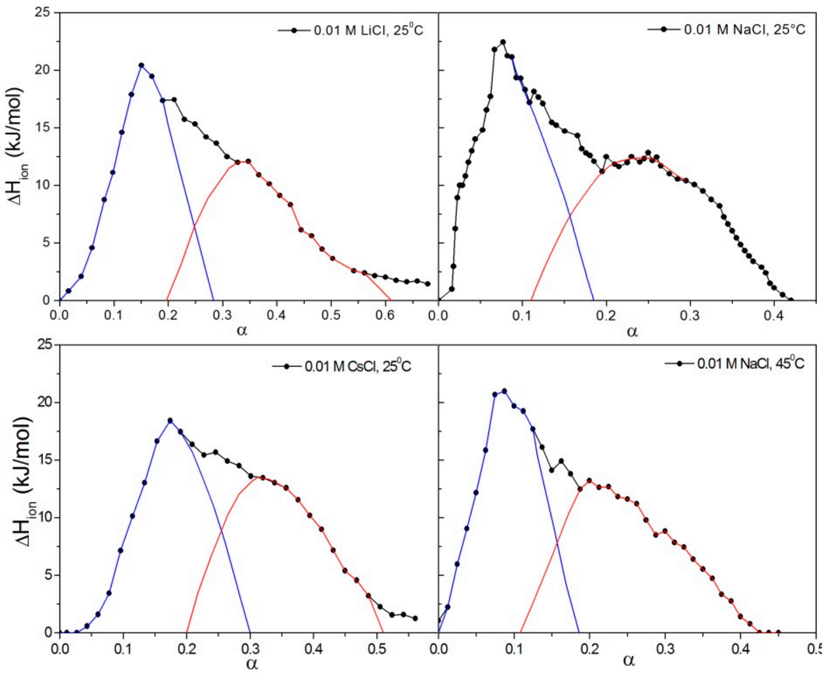

Figure S5. Deconvolution of calorimetric titration curves for iPMA in 0.01 M LiCl, NaCl and CsCl at 25 °C and for 0.01 M NaCl at 45 °C. The lines designate the two superimposed peaks in the low (blue line) and high (red line) α region that were integrated separately in order to calculate separate contributions of the conformational transition and intermolecular association to the total ΔH_{ion} value, *i.e.*, ΔH_{conf} (red line) and ΔH_{ass} (blue line) terms.

© 2016 by the authors; licensee MDPI, Basel, Switzerland. This article is an open access article distributed under the terms and conditions of the Creative Commons Attribution (CC-BY) license (http://creativecommons.org/licenses/by/4.0/).
